# Supplementary material for: GCfix: a fast and accurate fragment length-specific method for correcting GC bias in cell-free DNA
Source: Bioinformatics. 2025 May 12;41(6):btaf293. doi: 10.1093/bioinformatics/btaf293 (PMC12133280; doi:10.1093/bioinformatics/btaf293)
Supplement: btaf293_Supplementary_Data [file btaf293_supplementary_data.zip › Suppl_Figs.pdf]

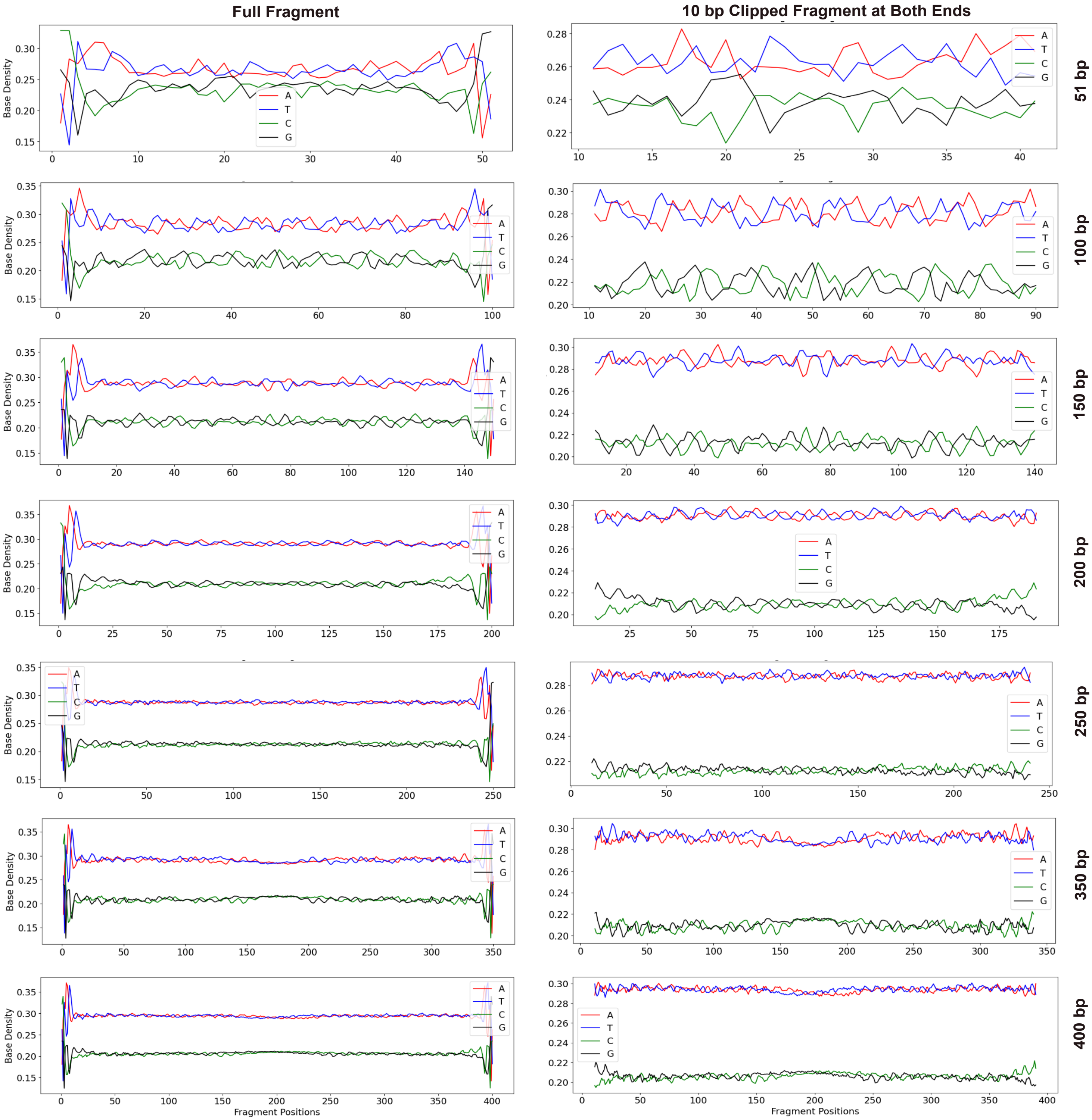

Suppl Fig S1: Base density in reference genome for full fragment GC context vs 10bp clipped (from both ends) fragment GC context for different fragment lengths of a healthy sample

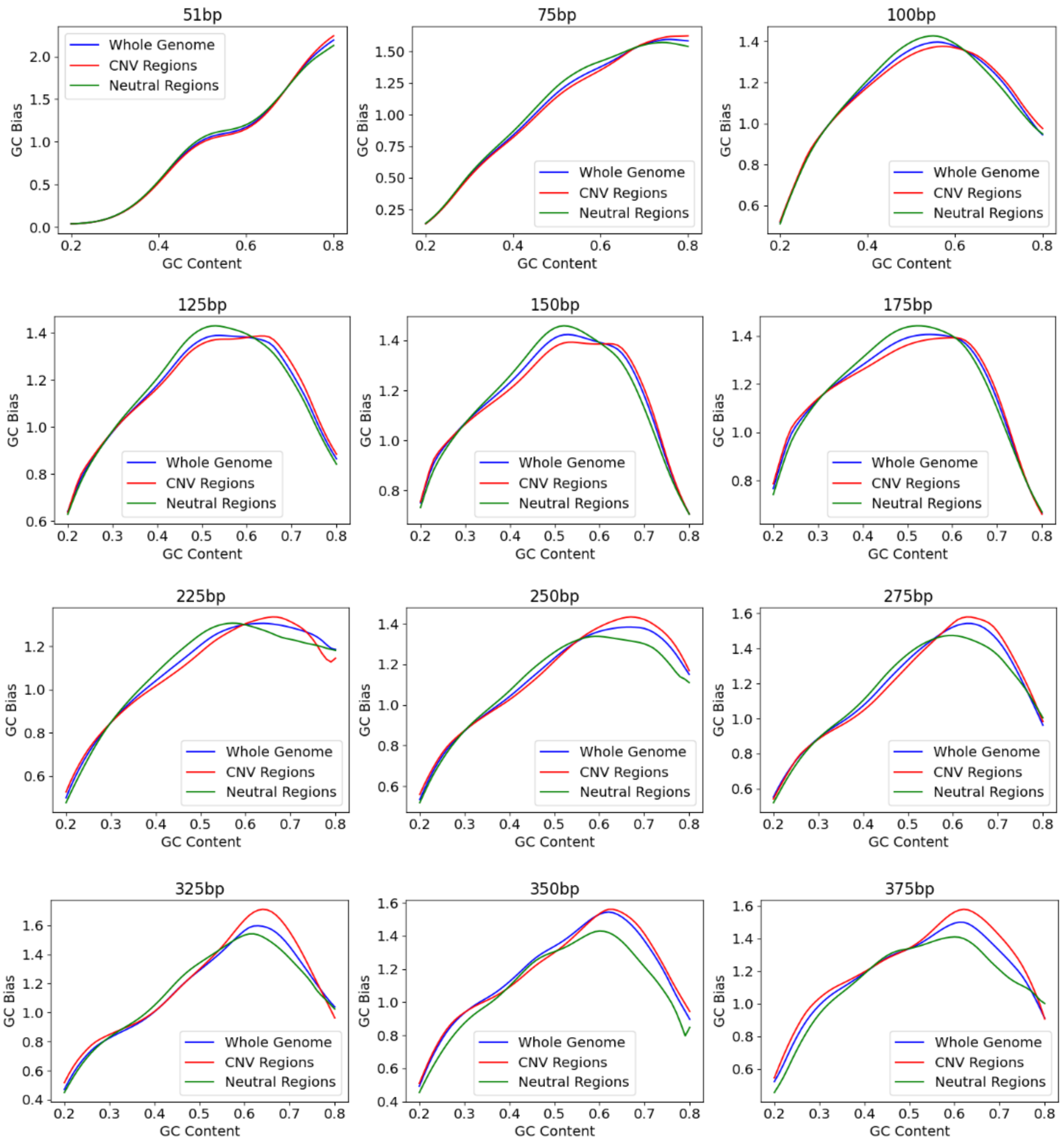

Suppl Fig S2: GC bias curve based on whole genome, neutral and copy number variant (CNV) regions for different fragment lengths of a colon cancer sample

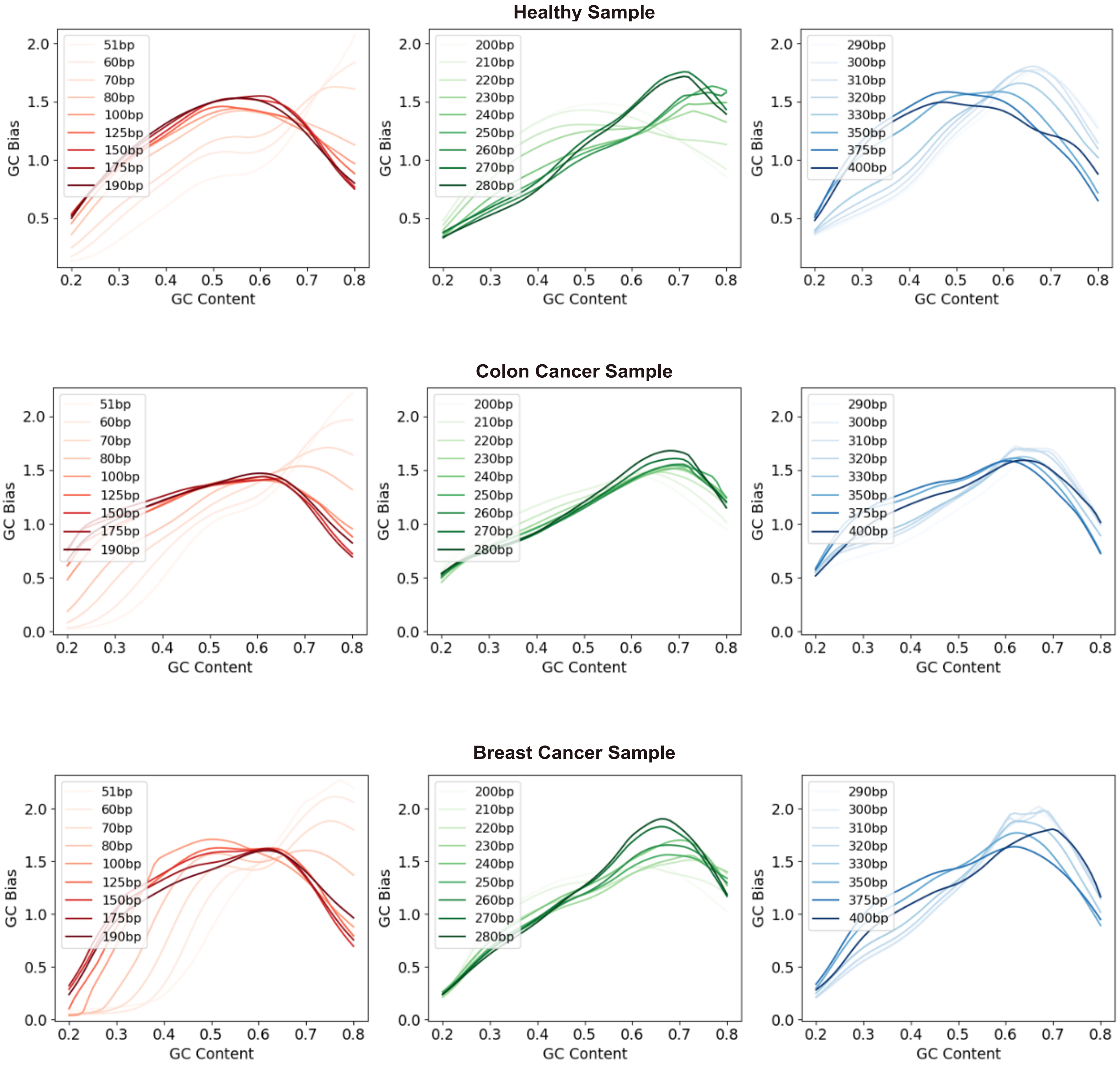

Suppl Fig S3: GC bias curves for different fragment lengths for healthy and cancer samples from different cohorts

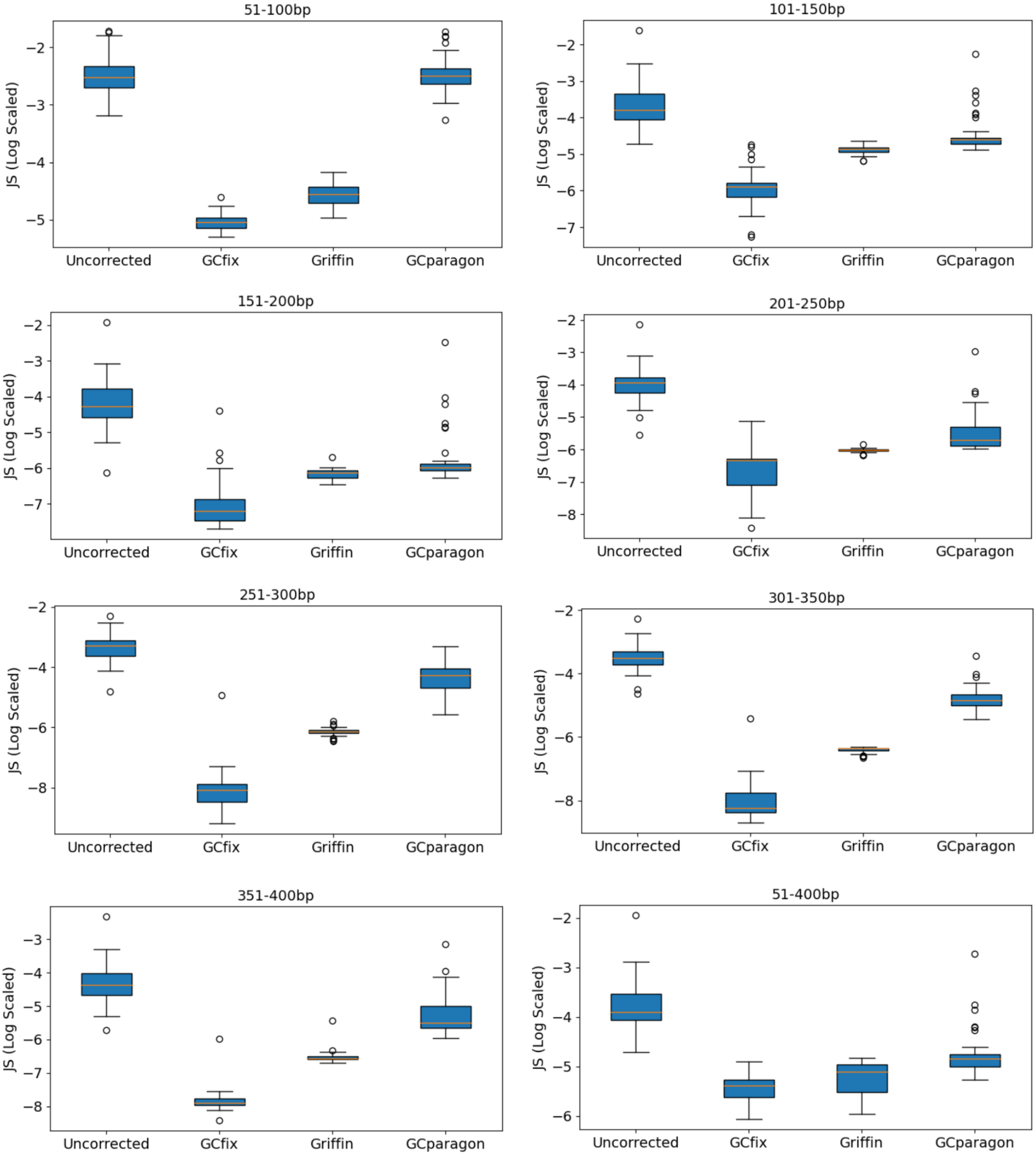

Suppl Fig S4: Sample JS divergence (log scaled) before and after correction (using different methods) from expected GC content fragment count density distribution based on different fragment length groups. 51 **deep WGS (>30X)** samples (both healthy and cancer) from 4 different cohorts have been used for each box plot.

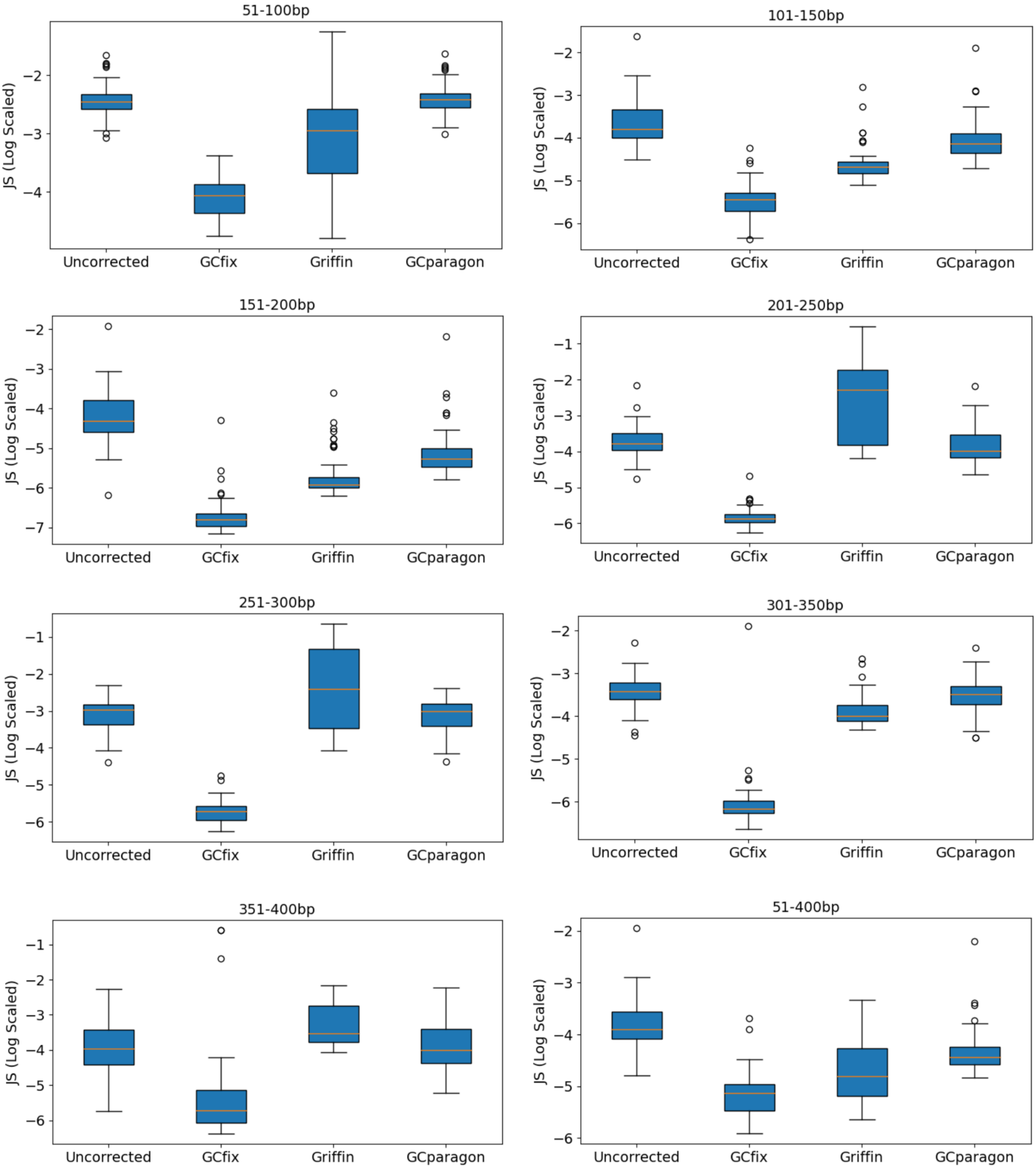

Suppl Fig S5: Sample JS divergence (log scaled) before and after correction (using different methods) from expected GC content fragment count density distribution based on different fragment length groups. 51 **ultra low pass WGS (~0.1X)** samples (both healthy and cancer) from 4 different cohorts have been used for each box plot.

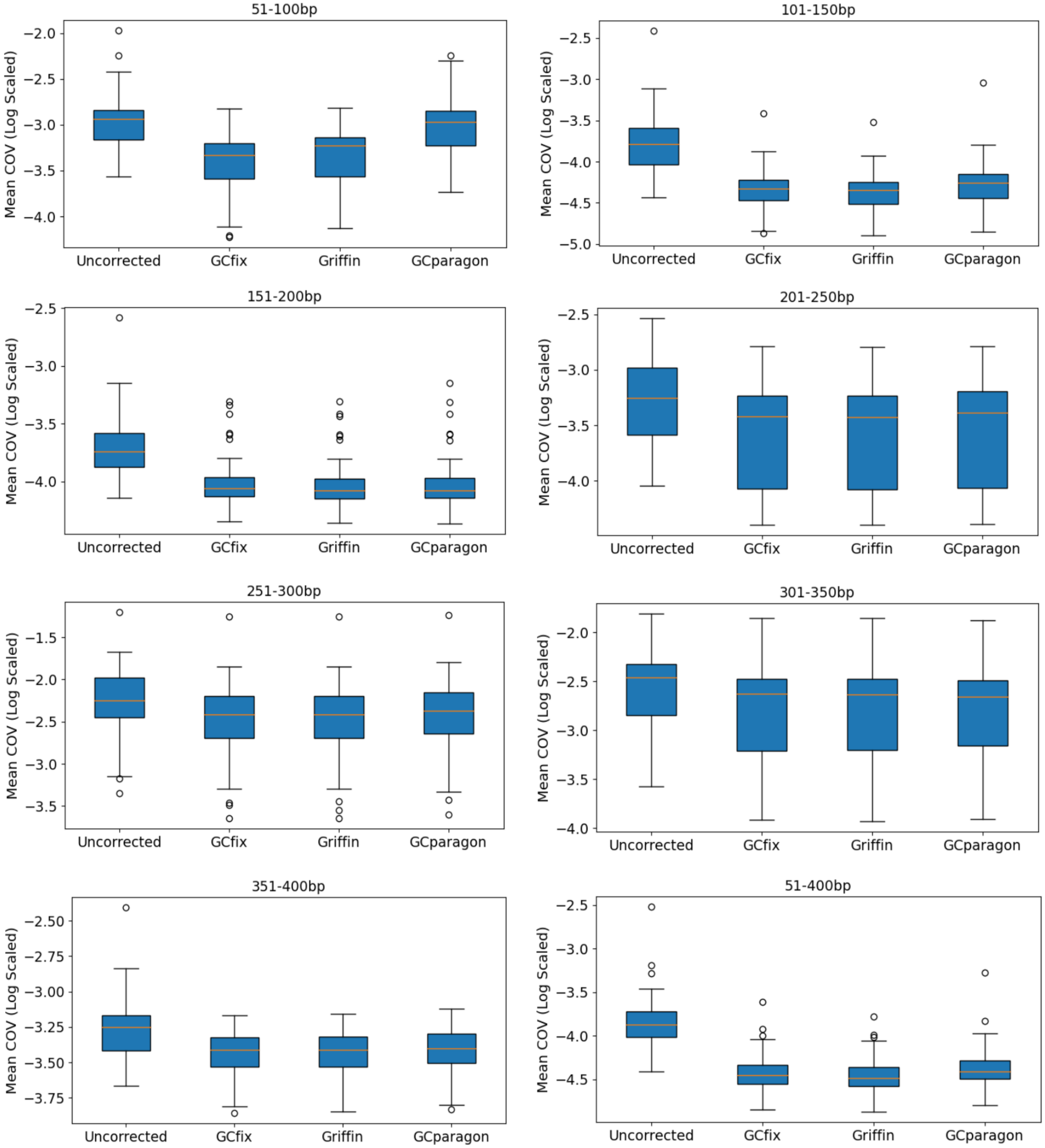

Suppl Fig S6: Sample mean coefficient of variation (log scaled) before and after correction (using different methods) based on different fragment length groups. 51 **deep WGS (>30X)** samples (both healthy and cancer) from 4 different cohorts have been used for each box plot.

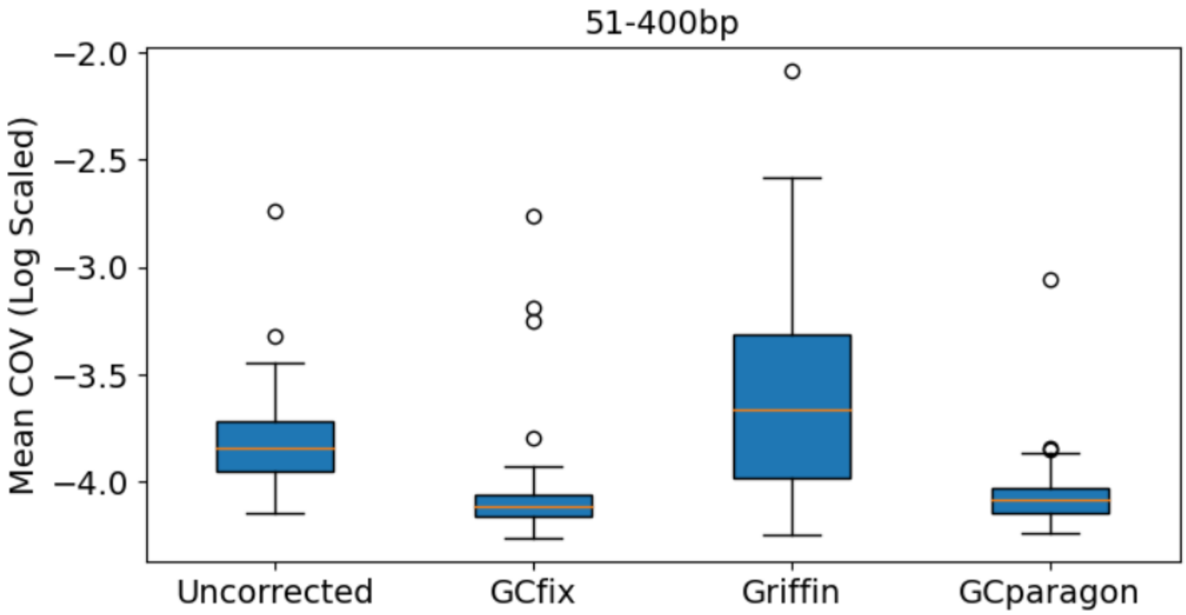

Suppl Fig S7: Sample mean coefficient of variation (log scaled) before and after correction (using different methods) based on all fragments of length 51-400bp. 51 **ultra low pass WGS (~0.1X)** samples (both healthy and cancer) from 4 different cohorts have been used for each box plot.

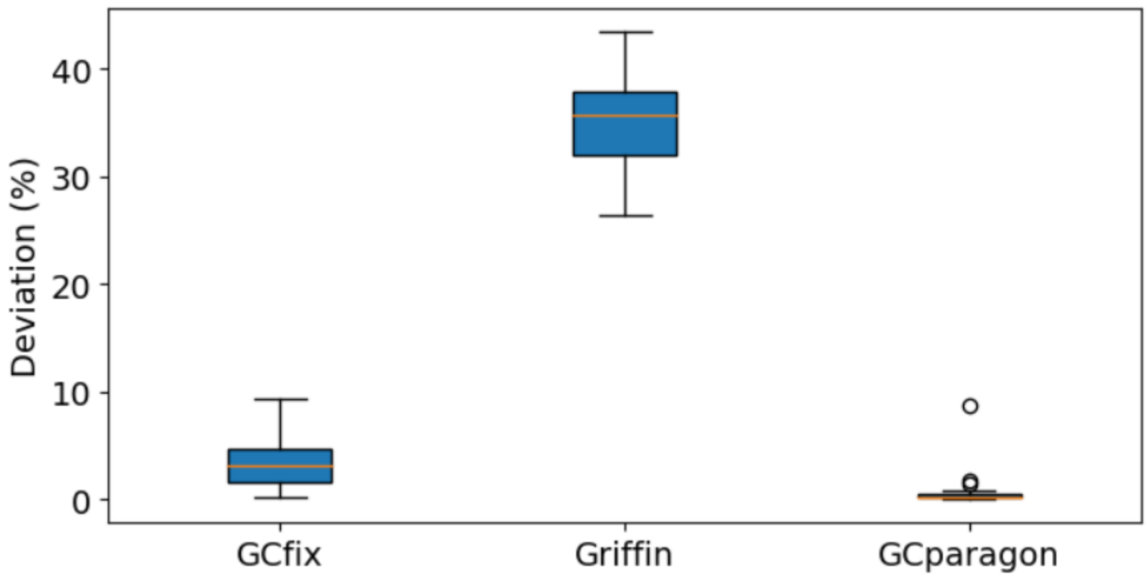

Suppl Fig S8: Deviation (%) in total fragment number after correction (using different methods) relative to the original uncorrected sample total fragment number using all samples (both healthy and cancer) from 4 different cohorts

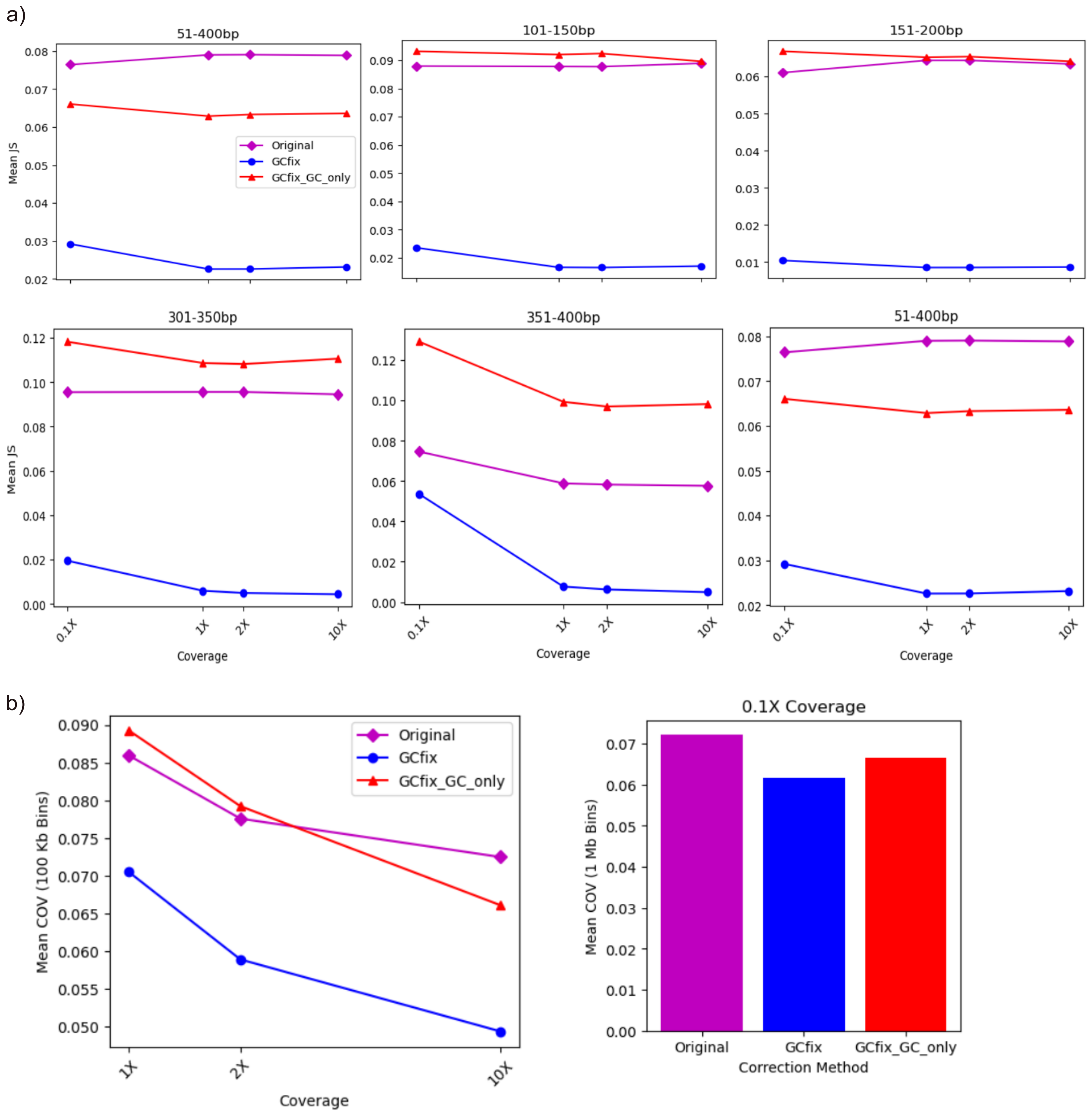

Suppl Fig S9: Comparing GCfix with length-agnostic GC%-only GCfix variant in terms of (a) JS divergence for different length groups and (b) coefficient of variation on samples of different coverages

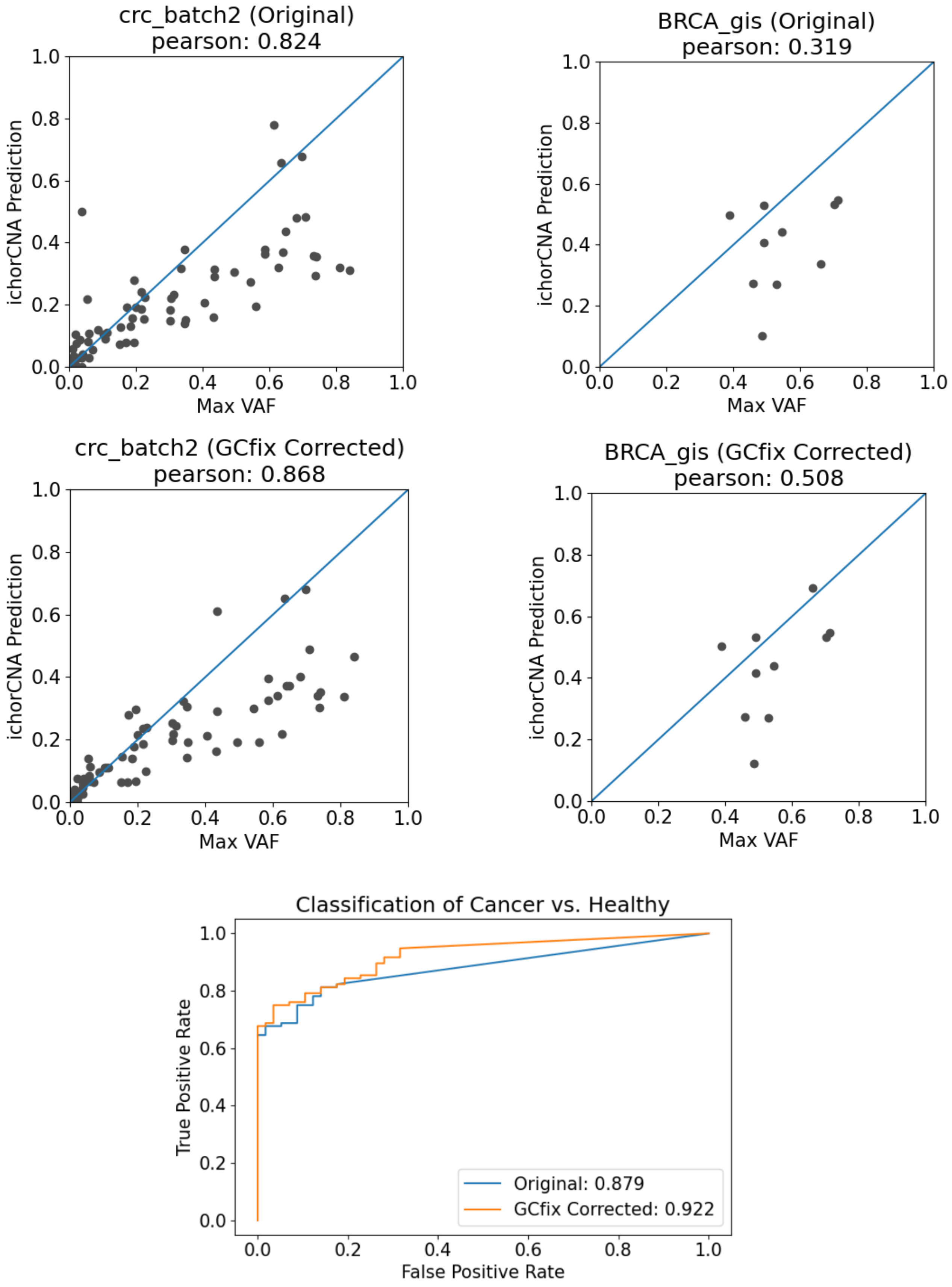

Suppl Fig S10: ichorCNA max VAF correlation and auROC plot before and after GCfix correction

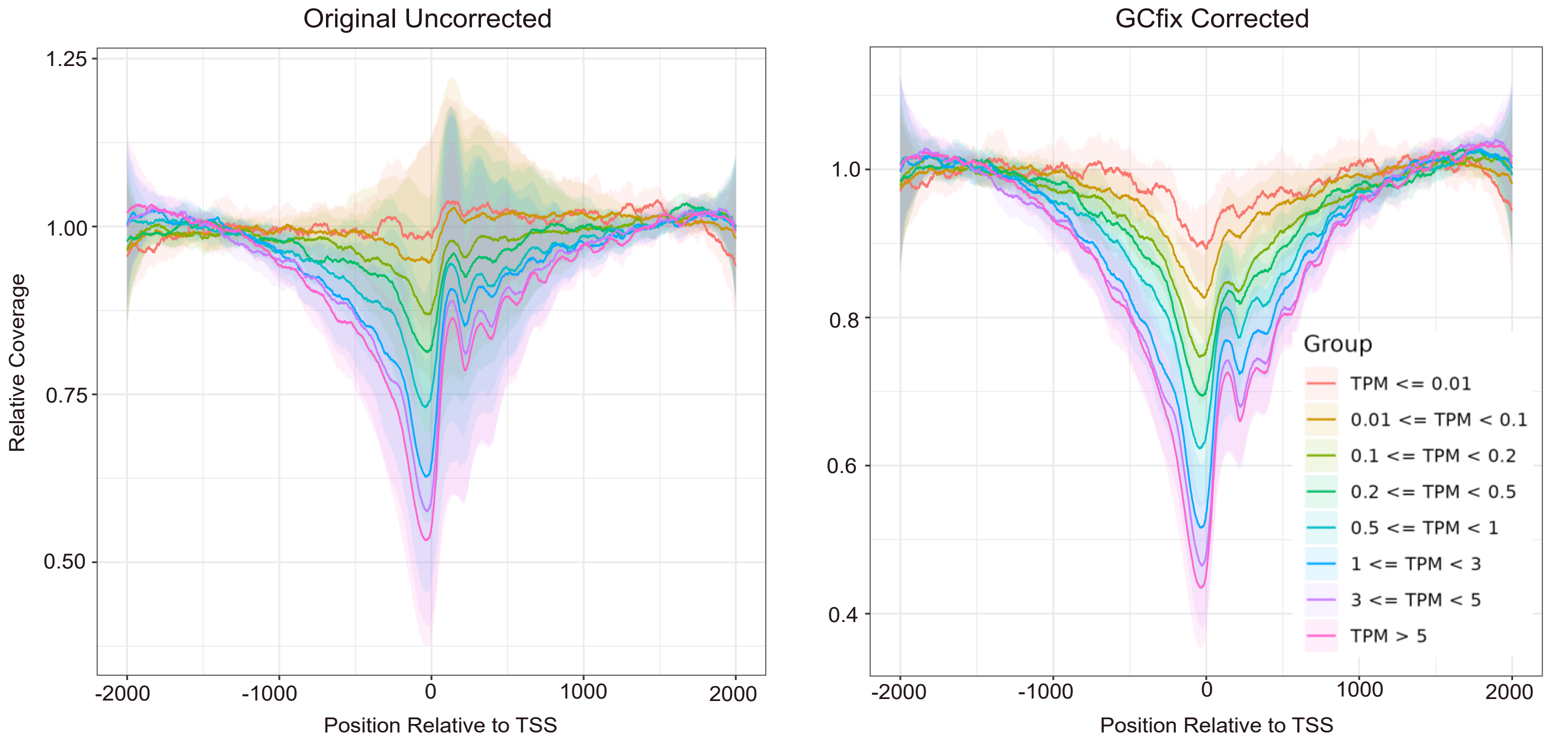

Suppl Fig S11: Relative coverage profile for different gene expression group nucleosome depleted regions (NDR) for the 17 deep WGS healthy samples before and after GCfix correction. GC correction significantly reduces the inter-sample variance in relative coverage profiles for the different gene expression groups.

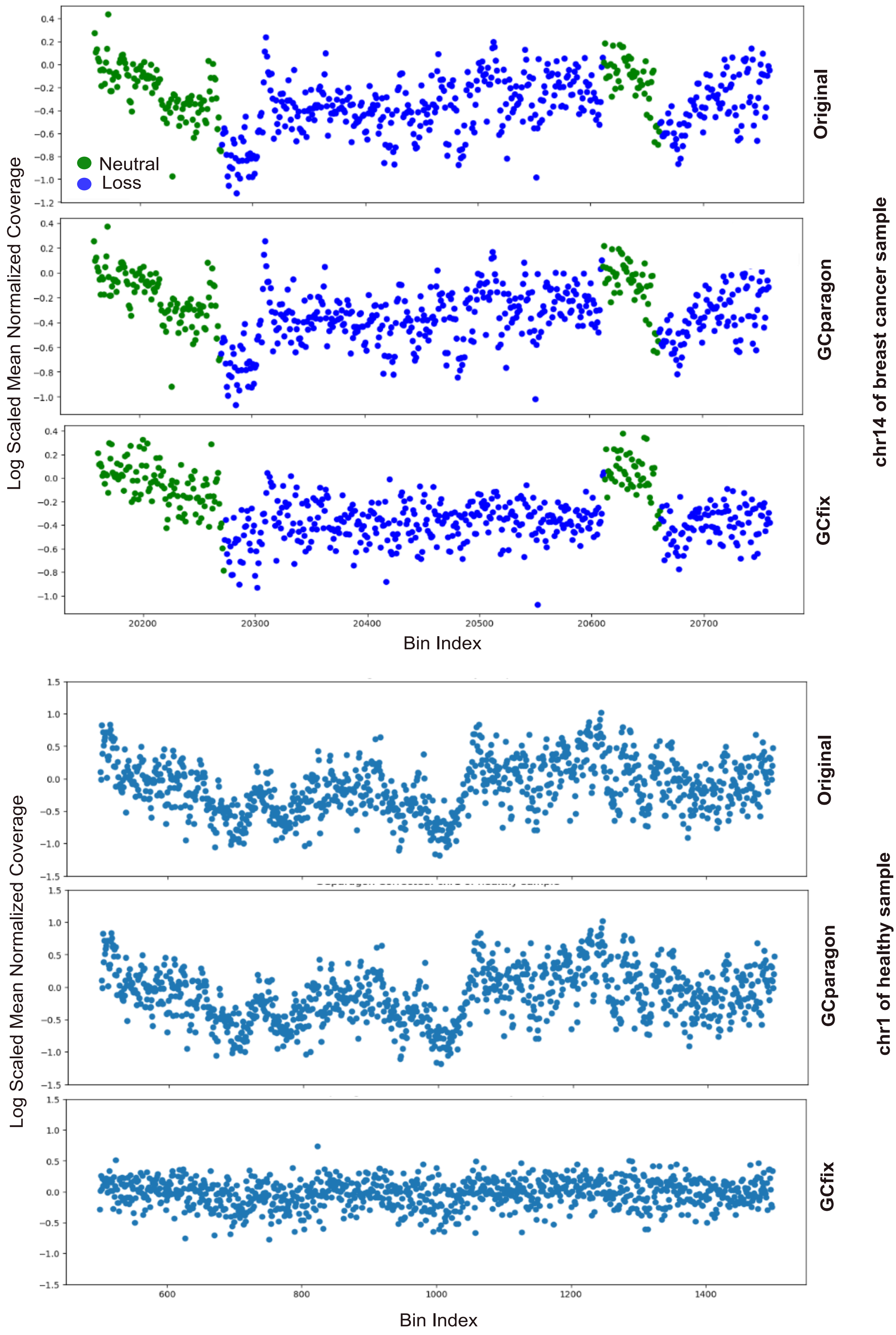

Suppl Fig S12: GCparagon does not correct for short fragment (51-100bp) coverage profile for cancer and healthy samples; GCfix does not have this limitation

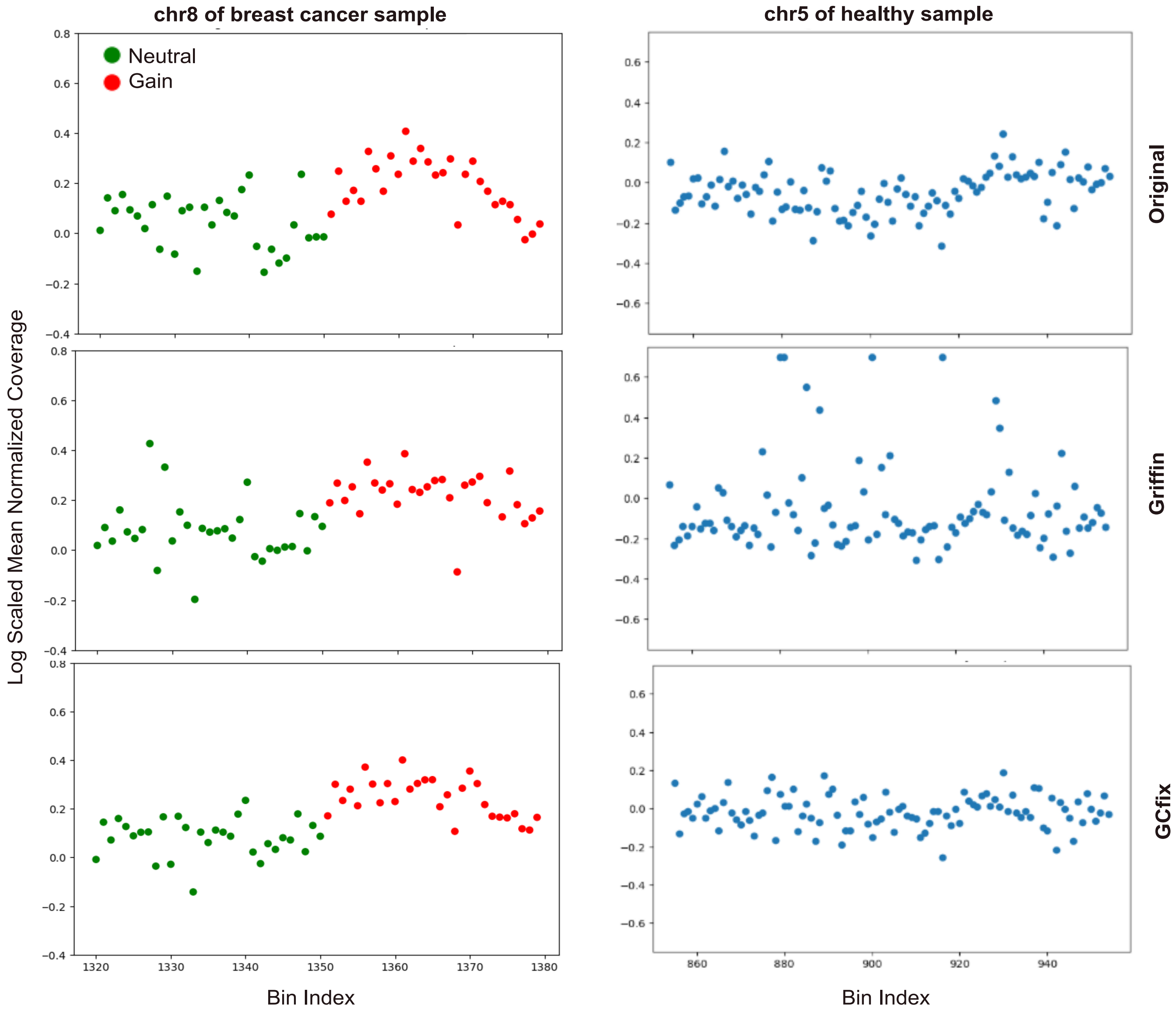

Suppl Fig S13: Griffin causes further disruption in the coverage profile for ultra low pass (~0.1X) WGS cancer and healthy samples. GCfix does not have this limitation.

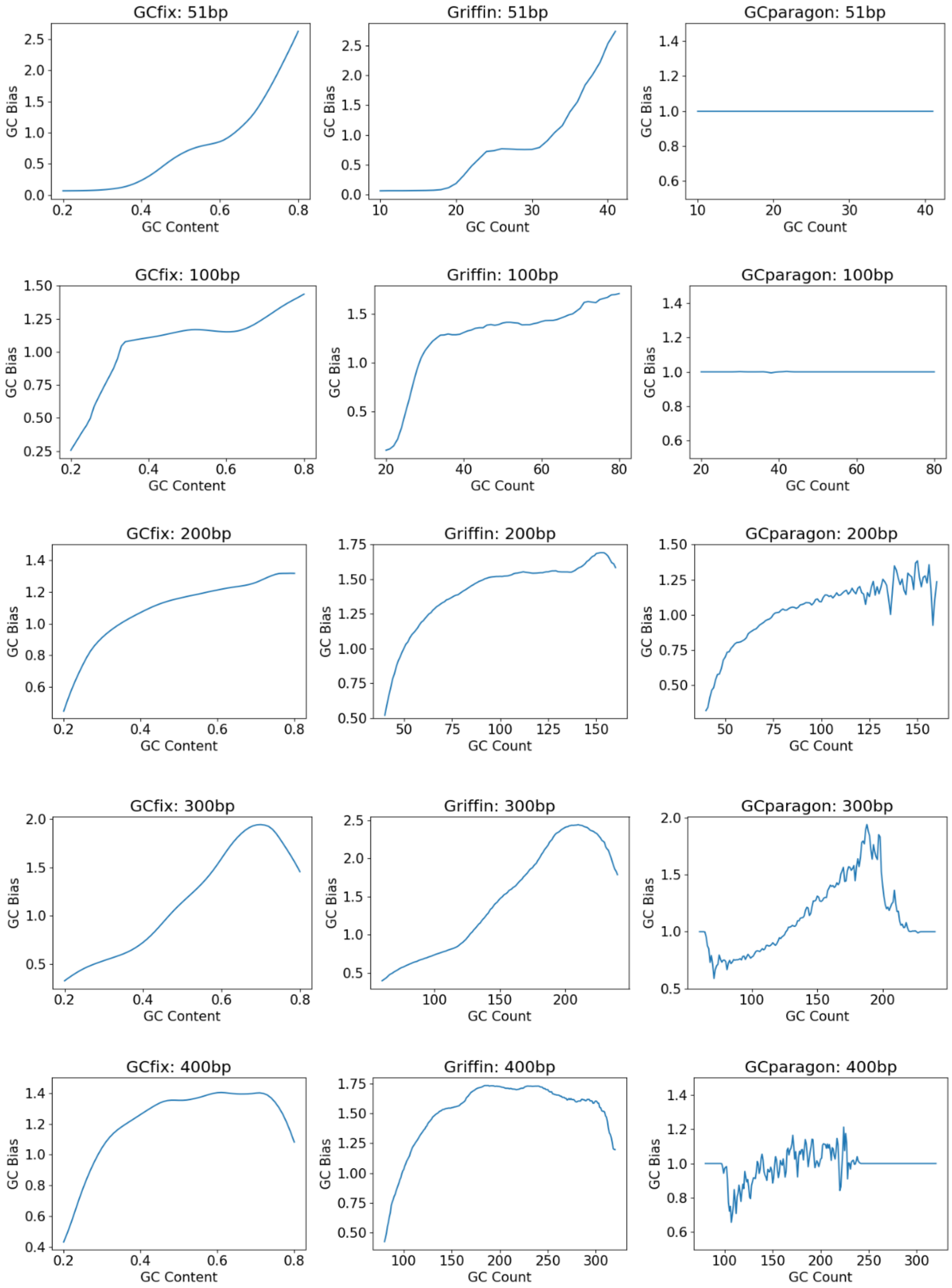

Suppl Fig S14: Comparing GC bias curves obtained from different GC correction methods for different fragment lengths of a healthy sample
